# A Study of Tourism Dynamics in Three Italian Regions Using a Nonautonomous Integrable Lotka–Volterra Model

**DOI:** 10.1371/journal.pone.0162559

**Published:** 2016-09-23

**Authors:** Alessandro Romano

**Affiliations:** LUISS Guido Carli, Rome, Italy; University of Rijeka, CROATIA

## Abstract

This article is a first application of an *integrable* nonautonomous Lotka–Volterra (LV) model to the study of tourism dynamics. In particular, we analyze the interaction in terms of touristic flows among three Italian regions. Confirming an hypothesis advanced by recent theoretical works, we find that these regions not only compete against each other, but at times they also proceed in mutualism. Moreover, the kind and the intensity of the interaction changes over time, suggesting that dynamic models can play a vital role in the study of touristic flows.

## Introduction

Over the last decades, tourism has become one of the largest and most profitable industries, thus attracting the attention of researchers belonging to different fields [1–3]. In this vein, a large body of literature has investigated how touristic locations compete against each other and what are the main drivers of their competitiveness [4, 5]. One fundamental characteristic of the “touristic product” is its inherent complexity, due to the number of stakeholders that can contribute to (or hinder) its value [6–9]. And indeed, influential scholars highlight that organizations operating within a touristic destination should coordinate to pursue a “collaborative advantage”, instead of a “competitive advantage”[10]. To further complicate the picture, an emerging strand of literature noted that the competitive strength of a given touristic destination crucially depends on how it interacts with other destinations [9]. However, quantitative studies investigating the kind and intensity of the interaction among touristic destinations that are grounded on rigorous mathematical models are lacking. This article is an attempt to fill this gap. We use the *integrable* nonautonomous LV model introduced in [11–13] to investigate the *kind* and the intensity of the interaction between three Italian regions: Campania, Puglia and Liguria.

The main findings of the paper can be summarized as follows. First, as predicted by the theoretical literature [9], the interaction among destinations is very nuanced and cannot be reduced to pure competition. Second, the kind and intensity of the interaction rapidly changes over time, and therefore the choice of a nonautonomous model is very appropriate. Third, the 2007 financial crisis only marginally changed the competition dynamics among the three destinations considered. A more detailed analysis of the results is presented in section Results.

## Model

LV models were created to capture the competitive struggle of *N* species in an established niche, and they have the very valuable property of being able to describe very nuanced forms of interaction besides the “standard” pure competition. Therefore, it should come to no surprise that LV models are extensively used in management studies to analyze firms’ competition in a given market [14–21]. In a few occasions, LV models have also been applied to the study of touristic flows [22]. However, all these models have two limitations: on the one hand, the analytic solutions are not known. On the other hand, all these models are *autonomous* (i.e. the interaction coefficients do not depend on time). When an analysis is performed using an autonomous model the competitive roles are automatically assumed to be constant over time. In other words, if a firm (or a touristic destination) is prey of another it will remain a prey for ever. This is a very strong limitation, because the interaction among locations is theorized to be fluid and always evolving [23].

We build on the model proposed by Marasco et al. [12, 13], because this model overcomes both problems: the analytic solutions are known and the interaction coefficients are explicitly dependent on time (i.e. we adopt a nonautonomous model). Knowing the analytic solutions is an advantage because it implies that LV coefficients no longer have to be estimated since they can directly be determined from the solutions. In turn, this makes the empirical analysis way easier and less data demanding. Incidentally, the analytic solution of this LV model *is* the logit model [11]. This shows that there is a very strong connection between the dynamic model proposed in this paper and the approach adopted by the mainstream literature on the study of touristic flows [24–26]. Importantly, in line with mainstream literature, this model features an outside good or option. As the outside option plays a relevant role, it is important to clarify this concept. In particular, the main feature of the outside option is its “passive” role in the niche considered. For example, let us assume that we are studying the interaction among beer producers. The outside good does not compete directly with the considered beer producers, but it is chosen only by those consumers that do not drink any kind of beer. In this example, an outside good in the market for beers could be wine. Similarly, in the case of tourism the outside location has a “passive” role and is chosen only by those tourists not interested in any of the inside locations. In other words, it has a *residual* market share.

### Lotka–Volterra Models and Competition Roles

The Lotka–Volterra system describing the evolution of *N* + 1-species is given by the following ordinary differential equations

$${\dot{x}}_{i}\left(t\right)=\underset{\text{logistic}\;\text{growth}}{\underset{︸}{a_{i}x_{i}\left(t\right)\left[1-\frac{x_{i}\left(t\right)}{k_{i}}\right]}}-\underset{\text{interaction}\;\text{with}\;\text{competitors}}{\underset{︸}{\sum_{j=0,j\neq i}^{N}c_{ij}x_{i}\left(t\right)x_{j}\left(t\right)}},\;i=0,\cdots ,N,$$ (1)

where *x*_*i*_(*t*) ≥ 0 represents the population size of the *i*th species at time *t*, ${\dot{x}}_{i}\left(t\right)=dx_{i}\left(t\right)/dt$, and the constant coefficients *a*_*i*_, *k*_*i*_, and *c*_*ij*_ are, respectively, the growth rates, the carrying capacity and the interaction coefficients of the *i*th specie. The carrying capacity of a specie in an environment is the maximum population size that the environment can sustain indefinitely. Therefore, here the concept of carrying capacity is different from what is generally intended in tourism research. In particular, because the data are presented in terms of touristic shares, the carrying capacity of each specie (or destination) can never exceed one. To study tourism dynamics using this framework the *N* + 1 species are the different locations competing for a scarce resource, namely tourists. We define the touristic share (*TS*_*i*_) of the *i*th location as the number of tourists visiting the *i*th location divided by the total number of tourists visiting all the locations considered. Formally, we denote *TS*_*i*_ as *x*_*i*_(*t*). The evolution of *TS*_*i*_ is determined by the intrinsic touristic growth rate *a*_*i*_ and the intraspecific competition rate between the *i*th and *j*th location *c*_*ij*_. Last, we recall that the maximum carrying capacity *k*_*i*_ = 1, as the sum of all the *TS*_*i*_ = 1. Then, Eq (1) become:

$${\dot{x}}_{i}\left(t\right)=x_{i}\left(t\right)\left[a_{i}-\sum_{j=0}^{N}d_{ij}x_{j}\left(t\right)\right],\;i=0,\cdots ,N,$$ (2)

where *d*_*ij*_ = *c*_*ij*_ for *j* ≠ *i*, and *d*_*ii*_ = *a*_*i*_.

Eqs (1) and (2) have been used by the scientific literature to describe different kinds of interactions. More precisely, following [14], the *kind* of interaction between *pairs* of competing entities is given by the signs of their interaction coefficients as indicated in Table 1.

**Table 1:** The competitive roles are given by P_*i*_(*t*) and P_*j*_(*t*).

*g*_*i*_	*g*_*j*_	type of interaction
+	+	pure competition
−	+	predator-prey
−	−	mutualism
−	0	commensalism
+	0	amensalism
0	0	neutralism

We assume that in Eq (2) *i* = 0 corresponds to the outside good or option, i.e., to the possibility that a traveler opts for a different location from the ones considered.

We recall that market shares must satisfy the following conditions

$$\begin{array}{c} 0\leq x_{i}\left(t\right)\leq 1,\;i=0,...,N,\\ \sum_{i=0}^{N}x_{i}\left(t\right)=1. \end{array}\forall t\geq t_{0}.$$ (3)

In view of Eq (3) (see [27]), the market share of the outside location is given by:

$$x_{0}\left(t\right)=1-\sum_{i=1}^{N}x_{i}\left(t\right).$$ (4)

We observe that system (2) is autonomous, therefore this systems can model only competitive roles that do not change over time.

### The Proposed Lotka–Volterra Model

To accurately describe touristic patterns, we rely on mathematical approaches that are extensively used by the mainstream literature on tourism research. More specifically, we look for a class of LV *nonautonomous* systems that has a strong link with approaches routinely used to describe touristic demand. Because a large part of the literature relies on the logit model, we build on the LV system developed in [12, 13]. And indeed, in [11] it is shown that the solution of this system *is* the logit model. We remark that it is extremely rare to know the analytical solutions of a *nonautonomous* LV model.

In other words, in natural sciences and in tourism research the two sides of the same coin were analyzed. On the one hand, natural scientists have been modeling competitive interactions using LV models. On the other hand, researchers in tourism have described touristic demand using the logit model. The proposed model uncovers the connection between the two approaches, because the latter is the analytical solution of the former.

Therefore, we consider the following class of LV nonautonomous systems

$${\dot{x}}_{i}\left(t\right)=x_{i}\left(t\right)\left[g_{i}\left(t\right)-\sum_{j=0}^{N}g_{j}\left(t\right)x_{j}\left(t\right)\right],\;i=0,\cdots ,N,$$ (5)

where *g*_*i*_(*t*) are integrable functions in any bounded interval. We note that this model has the structure of Eq (2), with the difference that the coefficients explicitly depend on time. This allows touristic locations to change their strategy and the kind of their competitive interaction over time. Further, the condition that the sum of *x*_*i*_(*t*) is equal to 1 for any value of *t* implies that Eq (5) for *i* = 0, which refers to the outside location *x*_0_(*t*), cannot be independent from the Eq (5) of the inside locations *x*_*i*_(*t*), for *i* = 1, …, *N*. Finally, the passive role of the outside location is ensured by the condition *g*_0_ = 0. In qualitative terms, the share of the outside location is the residual share not conquered by the inside locations. In conclusion, system (5) can be written in the form

$${\dot{x}}_{0}\left(t\right)=-x_{0}\left(t\right)\sum_{j=1}^{N}g_{j}x_{j}\left(t\right),$$ (6)

$${\dot{x}}_{i}\left(t\right)=x_{i}\left(t\right)\left[g_{i}\left(t\right)-\sum_{j=1}^{N}g_{j}\left(t\right)x_{j}\left(t\right)\right],\;i=1,\cdots ,N,$$ (7)

It is easy to check that the functions

$$x_{0}\left(t\right)=\frac{1}{1+\sum_{j=1}^{N}\text{exp}\left(f_{j}\left(t\right)\right)},$$ (8)

$$x_{i}\left(t\right)=\frac{\text{exp}\left(f_{i}\left(t\right)\right)}{1+\sum_{j=1}^{N}\text{exp}\left(f_{j}\left(t\right)\right)},\;i=1,\ldots ,N,$$ (9)

where

$${\dot{f}}_{i}\left(t\right)=g_{i}\left(t\right),$$ (10)

are a solution of systems (6) and (7) which satisfies condition (4) provided that we assume *f*_0_ = 0. In our model, the functions *f*_*i*_(*t*) are the utility functions of the different locations.

We conclude noting that evaluating the logarithms of *x*_*i*_(*t*) and *x*_0_(*t*), we obtain the following formula

$$f_{i}\left(t\right)=\ln\left(x_{i}\left(t\right)\right)-\ln\left(x_{0}\left(t\right)\right)\;i=1,...,N,$$ (11)

that gives the values of *f*_*i*_(*t*) in terms of the values of *x*_*i*_(*t*) and *x*_0_(*t*). In other words, this formula allows us to obtain the utility functions *f*_*i*_(*t*) using the experimental data on the touristic shares *x*_*i*_(*t*) and *x*_0_(*t*).

In view of Eq (9), we note that the share of each location is affected not only by its utility function, but also by the utility functions of the other competing locations. In particular, the share of the *i*th location increases when its utility function *f*_*i*_(*t*) increases and decreases when the utility function *f*_*j*_(*t*) of any other competitor increases. To this end, we note that

$$\begin{array}{c} \frac{dx_{i}}{df_{i}}>0\;\;\;\text{for}\;\text{all}\;i,\;\frac{dx_{i}}{df_{j}}<0\;\;\;\text{for}\;\text{all}\;j\neq i. \end{array}$$

How the share of each location varies in response to variations of one or more utility functions is evaluated through numerical simulations of Eq (9).

Eq (9) represent the generalized time-dependent logit demand function used in the standard market shares models proposed by McFadden and Kotler [28, 29].

In these models, Eq (9) expresses the utility derived by an individual choosing the alternative *i* out of a set of *N* available alternatives. In the linear logit models, the utility functions *f*_*i*_(*t*) are defined as a linear combination of suitable time-varying variables *V*_*h*_, *h* = 1, …, *M*, (characteristics of product, price, …), whereas in the nonlinear logit models the utility functions *f*_*i*_(*t*) are nonlinear functions of these variables [30, 31]. Among the relevant advantages of the latter there is the possibility to capture time discounting that is inherently non-linear [30, 31]. We recall that in the traditional literature the mean utility derived by a consumer from purchasing product *i* at time *t* is [32]

$$u_{i}\left(t\right)=-\alpha_{i}p_{i}\left(t\right)+\beta_{i}y_{i}\left(t\right)+\varepsilon_{i}\left(t\right),$$ (12)

where *y*_*i*_(*t*) is a function of observable characteristics of product *i*, *p*_*i*_(*t*) is the price, *ε*_*i*_(*t*) captures product- and time-specific shocks that are common to all consumers, and *α*_*i*_ and *β*_*i*_ are parameters. Therefore, in a framework of nonlinear logit models the utility functions Eq (12) are nonlinear in the variables *V*_*h*_ and depend non-linearly on time. It is easy to adapt this approach to our framework. Here, the utility functions describe the utility derived by a tourist of the *i*th destination, *p*_*i*_(*t*) represents the sum of monetary and non-monetary costs that the tourist must incur to visit the destination. In this vein, *p*_*i*_(*t*) decreases when the average prices in the location decrease or when it becomes easier to reach it (e.g. an airport is built in the proximity of the location). Last, *V*_*h*_ captures the characteristics of the destination and includes, among others, the cultural heritage, the proximity to the sea, the attractiveness of the beaches etc.

Importantly, we note that LV autonomous systems can only accommodate the linear logit model framework. The coefficients *g*_*i*_(*t*) in Eq (10) are constant if and only if the utility functions are linear combinations of the variables *V*_*h*_ and these variables depend linearly on time.

In detail, if we consider the utility functions in Eq (12) we obtain

$$g_{i}\left(t\right)=-\alpha_{i}{\dot{p}}_{i}\left(t\right)+\beta_{i}{\dot{y}}_{i}\left(t\right)+{\dot{\varepsilon }}_{i}\left(t\right),$$

Thus, the competitive roles are closely linked to the time variation of prices, of functions representing locations’ characteristics, and of terms capturing location- and time-specific shocks.

System (5) features all the main characteristics of an economic competition model. Yet, as stated above, it presents a relevant advantage over the existing alternatives. Instead of having to perform a numerical analysis, this model allows to solve the problem analytically. In this vein, the kind and the intensity of the interaction among competing entities can be derived with relative ease directly from the data on location shares. This allows to skip the complex and data-demanding step of estimating the parameters of the LV model.

An important step is to identify a functional form for the utility functions *f*_*i*_(*t*) that allows to mimic tourists’ behavior as accurately as possible.

In the logit demand models the utilities *f*_*i*_(*t*) are written as functions of observed location’s characteristics, price and demand parameters as in Eq (12). Consequently, the touristic share (represented by the choice probability relative to the destination) is given by the logit formula (9).

The problem is that estimating tourists’ utility functions is extremely hard. Nevertheless, thanks to the properties of the model proposed, it is possible to evaluate the utility functions *f*_*i*_(*t*) starting from the data on location shares. We proceed as follows: first, we introduce an outside location called “zero”. To not alter the results of the model we normalize its utility to 0. Therefore, Eq (11) holds. As stated above, because the utility of this outside location is set to 0 it has a merely passive role in the model. Second, we note that Eq (11) allow us to determine a discrete set of values for each utility function starting from historical data on location shares. Therefore, the indirect determination of the analytical form of these functions can be obtained by a fitting procedure. For any analytical form of these utility functions, if they are (*n* + 1)− times differentiable, it is possible to implement a fitting procedure starting from a polynomial model of degree *n*. In fact, from a mathematical point of view, this is equivalent to determine an analytical approximation of each utility function by the Taylor expansion of order *n*. When the utility functions are derived we can easily study the signs of the interaction coefficients and test the accuracy of the model.

### Data

We collect monthly data on presences in accommodation facilities of any kind (ranging from 5 stars hotels to camp sites) of both Italian and foreign tourists in the three Regions from 2003 to 2014. The data are obtained from the website of Osservatorio Nazionale del Turismo (National Observatory for Tourism, www.ontit.it) that in turns presents and organizes data gathered by the Istituto Nazionale di Statistica (ISTAT), and from the ISTAT itself (www.istat.it). ISTAT is the Italian main provider of official statistics. To have a better understanding of tourism patterns, the data are divided in pre-crisis (2003–2007) and crisis/post-crisis (2008–2014). This choice is dictated by the fitting procedure adopted. Because the data are fitted using a periodic function (Fourier series), the patterns described by the model are bound to repeat themselves in time. Therefore, analyzing together the data pre- and post- crisis could hide possible changes produced by the crisis in the competitive interaction among the touristic destinations. The data are aggregated in three different forms: All Year tourism (AY), Summer Tourism (ST) and Winter Tourism (WT). For AY the data on every month are considered and are then aggregated in three periods of four months (Jan-April, May-Aug, Sept-Dec). In the category ST only the four months characterized by the highest number of tourists are considered. For every location these months are June, July, August and September. Last, for WT the four months with the least tourists arrivals are considered. For every location the relevant months are January, February, November, and December. Importantly, all the data are presented in “touristic shares” and not in absolute values. Therefore, instead of using the absolute number of tourists as an input of the model, the share of tourists of each location is considered. For example, let us assume that in the month of January the total number of Italian tourists in the three locations is 50, with Campania, Puglia and Liguria hosting respectively 30, 10 and 10. The touristic share of Campania will be 0.6 (30/50), whereas that of Puglia and Liguria will be 0.2 (10/50). Besides allowing to follow the logit model, using touristic shares instead of absolute values has two important implications: (*i*) it allows to focus on the interaction between the three locations. In this vein, the study is not altered by macro events that affect the whole country. (*ii*) It puts the lens on the *relative* strength of the three locations. Because the fluctuations in the number of tourists across different months are common for all the locations, considering touristic shares allows to isolate the relative strength of each location *vis-à-vis* the others in each month of the year. In other words, fluctuations of touristic shares do not depend on the simple fact that in some months there is a systematically higher number of tourists than in others (i.e. seasonality [33, 34]). Instead, fluctuations in touristic shares reflect a change in the relative strength of the three competing locations. Last, to close the model it is necessary to add a fourth location that acts as an “outside good”. To not alter the mathematical results we introduce an imaginary location called “zero” that has a touristic share ≈ 0.

In principle, it would be interesting to analyze all Italian regions. However, for each region considered it must be included an additional differential equation. Thus, such an all-encompassing model would be composed of 20 differential equations making its description—especially via graphs—very complex and unsuited for a single Article. In this vein, for the sake of simplicity, we only consider Campania, Puglia and Liguria. Campania and Puglia were selected because they are the two most touristic regions of the Southern Italian peninsula. Moreover, the strong connection between the two Regions emerges also from official documents and authoritative research as there is often a special emphasis on them (e.g. [35]). Selecting two regions of the Southern Italian peninsula also allows testing an additional hypothesis. Over the recent years, various initiatives at an inter-regional level have been developed to promote tourism in South Italy on the ground that there could be synergies among Southern Italian regions. Among the most relevant, there is the Programma Operativo Interregionale (POIN) “Attrattori culturali, naturali e turismo” (a rough translation is “Interregional Operative Programme” called “Natural and Cultural Attractors and Tourism”). In this vein, it is interesting to uncover whether the interaction between Puglia and Campania has always been a pure competition or whether it is characterized by some degree of complementarity. Additionally, it is possible to study whether touristic flows in these areas depend on common factors like, for example, the brand “South Italy”. And indeed, if these regions are often in mutualism it can be inferred that an improvement of one also benefits the other via, maybe, an improvement of the perception of Southern Italy as a whole. This would be in line with the arguments advanced by a strand of literature emphasizing the importance of considering place branding from a multi-level perspective [23, 36].

Liguria is selected because it presents two relevant similarities with Campania, and therefore these regions might be considered relatively close competitors. First, the importance of the historic centers of the the main city of both regions, Napoli and Genova, has been official recognized by UNESCO. The whole historic center of Napoli has been included in the World Heritage List, whereas for Genova the UNESCO has included “Le Strade Nuove and the system of the Palazzi dei Rolli”. Second, both regions have a coastal area that attracts luxury tourism. Campania has the Amalfi Coast, the Sorrento Peninsula and the islands in the gulf of Napoli (especially Capri). Liguria has the Cinque Terre, Rapallo and Portofino.

## Results

Before describing the results of the analysis in detail, we remark that the measures of error considered—the Mean Absolute Percentage error (MAPE) and the Mean Standard Error (MSE)—indicate that the model can accurately describe touristic dynamics. In particular, as reported in Tables 2 and 3 the model is “highly accurate” in 33 instances out of 36. Following [37], we consider the model to be “highly accurate” when MAPE < 10. For all the Figures in this section Campania is depicted in red, Puglia in Black and Liguria in Blue.

**Table 2:** MAPE < 10% = Highly Accurate.

Period	Campania	Puglia	Liguria
For AY 2003–2007	1.32723	6.05118	2.68658
For AY 2008–2014	3.7053	7.01377	3.75666
Ita AY 2003–2007	2.07783	4.92433	1.56125
Ita AY 2008–2014	3.57867	5.12716	4.12766
For Summer 2003–2007	2.85351	9.83704	2.40577
For Summer 2008–2014	4.87972	8.50081	4.84916
Ita Summer 2003–2007	5.57394	6.89578	3.35232
Ita Summer 2008–2014	7.34159	8.78914	5.87928
For Winter 2003–2007	3.61446	12.2601	6.41817
For Winter 2008–2014	6.23283	9.69202	7.08751
Ita Winter 2003–2007	8.77974	9.44673	11.3182
Ita Winter 2008–2014	7.82938	9.53869	13.727

**Table 3:** MSE.

Period	Campania	Puglia	Liguria
For AY 2003–2007	0.000104868	0.0000505844	0.00013459
For AY 2008–2014	0.000504273	0.00013257	0.000234297
Ita AY 2003–2007	0.000126746	0.000145052	0.0000432255
Ita AY 2008–2014	0.000394028	0.00029538	0.000328793
For Summer 2003–2007	0.000407005	0.000183359	0.0000752519
For Summer 2008–2014	0.000974386	0.000235529	0.000364176
Ita Summer 2003–2007	0.000567907	0.000877302	0.000192196
Ita Summer 2008–2014	0.000965166	0.00169246	0.000433005
For Winter 2003–2007	0.000721359	0.000247976	0.00051916
For Winter 2008–2014	0.00149553	0.000342023	0.000905052
Ita Winter 2003–2007	0.00184518	0.000417932	0.00324756
Ita Winter 2008–2014	0.00154761	0.000721461	0.00405003

### Foreign Tourists All Year

Let us start the analysis from the data on Foreign Tourists AY. The touristic shares of the three locations and the outside location for the periods 2003–2007 and 2008–2014 are reported in Tables 4 and 5:

**Table 4:** Touristic Shares AY for Foreign Tourists 2003–2007.

Period	Campania	Puglia	Liguria	Zero
2003–T1	0.568536	0.0784408	0.35302	3. ‵*^∧^ - 6
2003—T2	0.577682	0.127623	0.294692	3. ‵*^∧^ - 6
2003—T3	0.648601	0.089012	0.262384	3. ‵*^∧^ - 6
2004—T1	0.598317	0.08193	0.31975	3. ‵*^∧^ - 6
2004—T2	0.586002	0.126107	0.287888	3. ‵*^∧^ - 6
2004—T3	0.66051	0.0846536	0.254833	3. ‵*^∧^ - 6
2005—T1	0.604878	0.0931272	0.301992	3. ‵*^∧^ - 6
2005—T2	0.588655	0.122517	0.288825	3. ‵*^∧^ - 6
2005—T3	0.652361	0.0968025	0.250833	3. ‵*^∧^ - 6
2006–T1	0.566767	0.0940344	0.339196	3. ‵*^∧^ - 6
2006—T2	0.584816	0.118217	0.296963	3. ‵*^∧^ - 6
2006—T3	0.638115	0.100668	0.261213	3. ‵*^∧^ - 6
2007—T1	0.585844	0.107094	0.307059	3. ‵*^∧^ - 6
2007—T2	0.580679	0.124825	0.294493	3. ‵*^∧^ - 6
2007–T3	0.6413	0.0991593	0.259537	3. ‵*^∧^ - 6

**Table 5:** Touristic Shares AY for Foreign Tourists 2008–2014.

Period	Campania	Puglia	Liguria	Zero
2008—T1	0.558254	0.108908	0.332835	3. ‵*^∧^ - 6
2008—T2	0.547332	0.135855	0.31681	3. ‵*^∧^ - 6
2008—T3	0.603072	0.119446	0.277479	3. ‵*^∧^ - 6
2009—T1	0.523096	0.123377	0.353523	3. ‵*^∧^ - 6
2009—T2	0.532033	0.136678	0.331285	3. ‵*^∧^ - 6
2009—T3	0.592745	0.118547	0.288705	3. ‵*^∧^ - 6
2010—T1	0.515377	0.121841	0.362778	3. ‵*^∧^ - 6
2010—T2	0.532841	0.145461	0.321694	3. ‵*^∧^ - 6
2010—T3	0.589577	0.125999	0.284421	3. ‵*^∧^ - 6
2011—T1	0.531515	0.134578	0.333904	3. ‵*^∧^ - 6
2011—T2	0.526439	0.153099	0.320459	3. ‵*^∧^ - 6
2011—T3	0.592227	0.132561	0.275209	3. ‵*^∧^ - 6
2012—T1	0.560523	0.117949	0.321525	3. ‵*^∧^ - 6
2012–T2	0.499108	0.169915	0.330974	3. ‵*^∧^ - 6
2012–T3	0.587877	0.137738	0.274381	3. ‵*^∧^ - 6
2013–T1	0.541132	0.133433	0.325432	3. ‵*^∧^ - 6
2013–T2	0.485794	0.165607	0.348596	3. ‵*^∧^ - 6
2013–T3	0.558665	0.143023	0.298308	3. ‵*^∧^ - 6
2014–T1	0.573684	0.125922	0.300391	3. ‵*^∧^ - 6
2014—T2	0.482957	0.16989	0.34715	3. ‵*^∧^ - 6
2014—T3	0.530525	0.153015	0.316457	3. ‵*^∧^ - 6

We first analyze the data relative to 2003–2007. The data reveal a constant pattern of fluctuations in the touristic shares of the three locations, with Campania dominating in *t*_3_, Puglia performing relatively better in *t*_2_, and Liguria having its golden period in *t*_1_. Using Eq (17) we obtain a set of discrete values of the utility functions characterized by a similar pattern of fluctuations. Therefore, the data suggest that the functional form of the utility functions can be described by the Fourier expansion $a+b\sin(\frac{\pi t}{T})+c\cos(\frac{\pi t}{T})$. We use the routine *FindFit* of the software *Mathematica* to fit the data.

**Fig 1:**
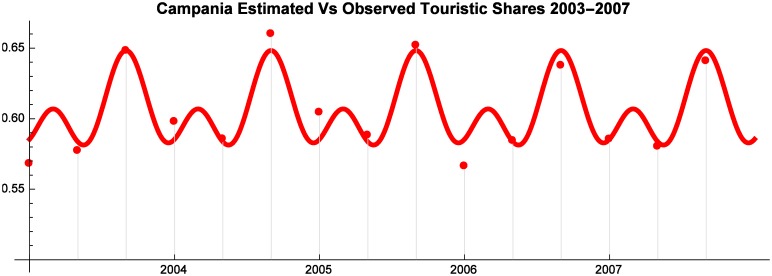
Campania Estimated vs Observed Touristic Shares 2003–2007.

**Fig 2:**
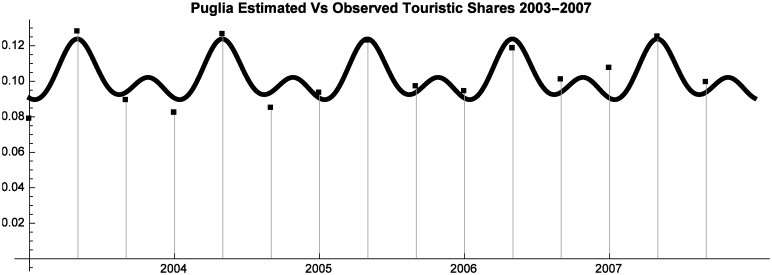
Puglia Estimated vs Observed Touristic Shares 2003–2007.

**Fig 3:**
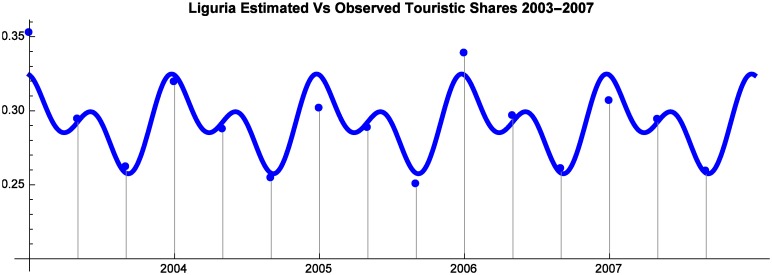
Liguria Estimated vs Observed Touristic Shares 2003–2007.

As shown by Figs 1, 2 and 3 the utility functions offer an accurate approximation of observed Market Shares.

**Fig 4:**
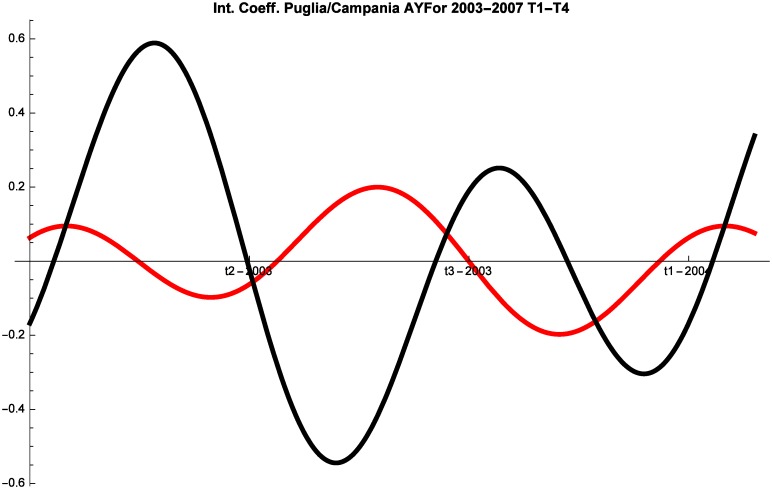
Campania (red) vs Puglia (black) Competitive Roles AYFor 2003–2007, T1–T4.

**Fig 5:**
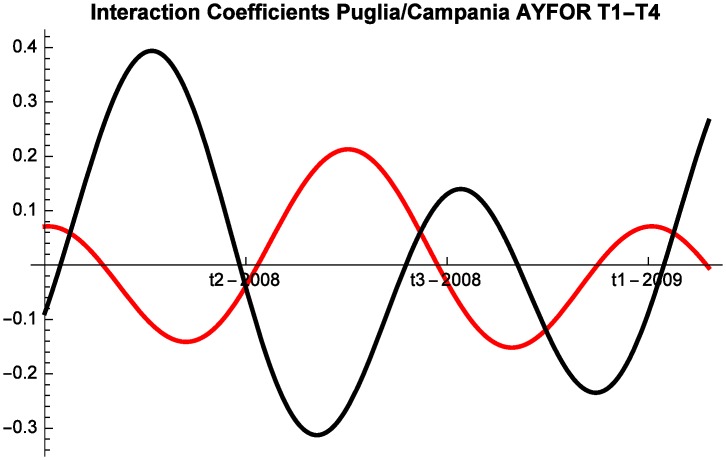
Campania (red) vs Puglia (black) Competitive Roles AYFor 2008–2014, T1–T4.

Let us now study the competitive interaction of the three destinations. Interestingly, we note that the locations frequently change their competitive behavior during the year. For example, the interaction coefficients of Campania and Puglia are represented by the Fig 4:

Let us now turn to the 2008–2014 time interval. Also during this period the destinations frequently change competitive interaction. The most interesting insight is that the interaction dynamics are very similar in the two time intervals, suggesting that the crisis did not significantly alter the competitive interaction for this segment of the market (AYFor). Additionally, counterintuitively, the intensity of the competition is lower during and following the crisis. And indeed, the competition coefficients of Puglia (and Liguria) generally have smaller absolute values between 2008 and 2014 (see Fig 5).

While a detailed discussion of the findings of the model will be performed in the next section, two preliminary remarks should be made. First, as predicted by the theoretical literature [9], the interaction between touristic locations cannot be reduced to pure competition. For example, the interaction between Campania and Puglia is very nuanced and the regions mostly stand in a predator-prey relationship in which they alternate roles. But they also engage in pure competition and at times proceed in mutualism. Second, the interaction between locations is inherently dynamic and hence any absolute claim on the competitive or cooperative nature of the interaction is bound to be inaccurate. At times destinations compete, whereas at other times they might proceed in mutualism or commensalism.

### Italian Tourists All Year

We now move to analyze the behavior of Italian tourists. The location shares are indicated in Tables 6 and 7

**Table 6:** Touristic Shares AY for Italian Tourists 2003–2007.

Period	Campania	Puglia	Liguria	Zero
2003—T1	0.347875	0.147043	0.505079	3. ‵*^∧^ - 6
2003—T2	0.322902	0.359338	0.317757	3. ‵*^∧^ - 6
2003—T3	0.518884	0.209738	0.271375	3. ‵*^∧^ - 6
2004—T1	0.374021	0.135007	0.490969	3. ‵*^∧^ - 6
2004—T2	0.32796	0.361046	0.310991	3. ‵*^∧^ - 6
2004—T3	0.518171	0.201515	0.280311	3. ‵*^∧^ - 6
2005—T1	0.35133	0.154094	0.494573	3. ‵*^∧^ - 6
2005—T2	0.313956	0.376991	0.30905	3. ‵*^∧^ - 6
2005—T3	0.500166	0.230933	0.268898	3. ‵*^∧^ - 6
2006—T1	0.353431	0.155199	0.491367	3. ‵*^∧^ - 6
2006—T2	0.326095	0.356258	0.317643	3. ‵*^∧^ - 6
2006—T3	0.484276	0.233981	0.28174	3. ‵*^∧^ - 6
2007—T1	0.344355	0.16607	0.489572	3. ‵*^∧^ - 6
2007—T2	0.324872	0.380806	0.294319	3. ‵*^∧^ - 6
2007—T3	0.482338	0.241179	0.27648	3. ‵*^∧^ - 6

**Table 7:** Touristic Shares AY for Italian Tourists 2008–2014.

Period	Campania	Puglia	Liguria	Zero
2008—T1	0.346642	0.172276	0.481078	3. ‵*^∧^ - 6
2008—T2	0.311587	0.398641	0.289769	3. ‵*^∧^ - 6
2008—T3	0.47164	0.259484	0.268873	3. ‵*^∧^ - 6
2009—T1	0.358239	0.186907	0.454852	3. ‵*^∧^ - 6
2009—T2	0.303695	0.40743	0.288872	3. ‵*^∧^ - 6
2009—T3	0.465335	0.261487	0.273175	3. ‵*^∧^ - 6
2010—T1	0.363766	0.193567	0.442664	3. ‵*^∧^ - 6
2010—T2	0.307243	0.41488	0.277874	3. ‵*^∧^ - 6
2010—T3	0.469998	0.272928	0.257071	3. ‵*^∧^ - 6
2011—T1	0.36107	0.205473	0.433454	3. ‵*^∧^ - 6
2011—T2	0.307378	0.420429	0.272189	3. ‵*^∧^ - 6
2011—T3	0.479213	0.264323	0.256461	3. ‵*^∧^ - 6
2012—T1	0.378366	0.194935	0.426695	3. ‵*^∧^ - 6
2012—T2	0.301702	0.428788	0.269507	3. ‵*^∧^ - 6
2012–T3	0.452922	0.30073	0.246344	3. ‵*^∧^ - 6
2013–T1	0.385428	0.204526	0.410043	3. ‵*^∧^ - 6
2013–T2	0.304368	0.443884	0.251745	3. ‵*^∧^ - 6
2013–T3	0.409667	0.322027	0.268303	3. ‵*^∧^ - 6
2014–T1	0.420576	0.186018	0.393403	3. ‵*^∧^ - 6
2014–T2	0.302718	0.437628	0.259651	3. ‵*^∧^ - 6
2014–T3	0.417387	0.305877	0.276732	3. ‵*^∧^ - 6

There are two important points that can be noted. First, compared to the foreign tourists segment, Campania has a lower touristic share in each period for both time intervals considered (2003–2007, 2008–2014). This implies that foreign tourists appreciate Campania as an all-around touristic destination more than Italian tourists. Second, the share of Puglia is higher for Italian tourists than for foreign tourists in each period, in particular during summer. This suggests that Puglia has not yet built a strong reputation outside the Italian borders, especially for historical and cultural tourism.

**Fig 6:**
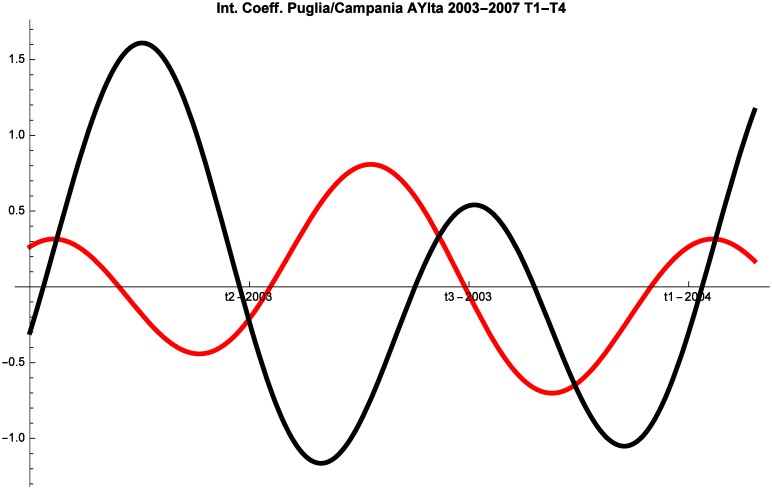
Campania (red) vs Puglia (black) Competitive Roles AYIta 2003–2007.

**Fig 7:**
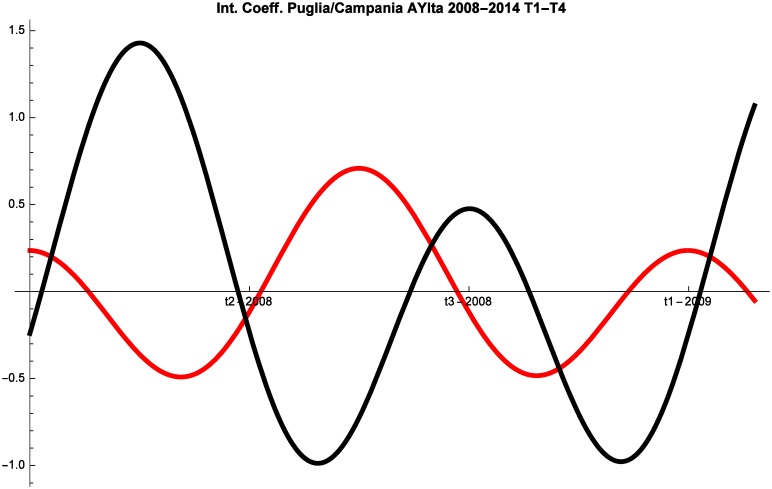
Campania (red) vs Puglia (black) Competitive Roles AYIta 2008–2014.

We follow the same routine also in this case and we note that the three regions change their competitive behavior at a very fast pace and alternate periods of pure competition and predator-prey with periods in which they proceed in mutualism. It is remarkable that the interaction between Puglia and Campania is basically identical in the two periods analyzed (see Figs 6 and 7).

### Tourists Summer: Italian and Foreign Tourists

While it was reasonable to expect fluctuations over an entire year, the constant fluctuations *within* the same season were less predictable. As indicated in Tables 8 and 9, fluctuations can be observed also when focusing only on summer months for both 2003–2007 and 2008–2014.

**Table 8:** Touristic Shares Summer for Italian Tourists 2003–2007.

Period	Campania	Puglia	Liguria	Zero
Jun—2003	0.350401	0.285553	0.364043	3. ‵*^∧^ - 6
Jul—2003	0.316622	0.392315	0.29106	3. ‵*^∧^ - 6
Aug—2003	0.291754	0.417677	0.290566	3. ‵*^∧^ - 6
Sept—2003	0.475538	0.242904	0.281555	3. ‵*^∧^ - 6
Jun—2004	0.351018	0.291615	0.357363	3. ‵*^∧^ - 6
Jul—2004	0.304513	0.403121	0.292364	3. ‵*^∧^ - 6
Aug—2004	0.306079	0.412139	0.281778	3. ‵*^∧^ - 6
Sept—2004	0.503217	0.208353	0.288426	3. ‵*^∧^ - 6
Jun—2005	0.339606	0.296849	0.363543	3. ‵*^∧^ - 6
Jul—2005	0.30462	0.405849	0.289528	3. ‵*^∧^ - 6
Aug—2005	0.289451	0.435799	0.274747	3. ‵*^∧^ - 6
Sept—2005	0.474722	0.262914	0.262361	3. ‵*^∧^ - 6
Jun—2006	0.35325	0.282059	0.364688	3. ‵*^∧^ - 6
Jul—2006	0.316288	0.382939	0.30077	3. ‵*^∧^ - 6
Aug—2006	0.297001	0.414889	0.288108	3. ‵*^∧^ - 6
Sept—2006	0.465127	0.266135	0.268735	3. ‵*^∧^ - 6
Jun—2007	0.350266	0.312868	0.336863	3. ‵*^∧^ - 6
Jul—2007	0.311908	0.410534	0.277555	3. ‵*^∧^ - 6
Aug—2007	0.303023	0.434375	0.262599	3. ‵*^∧^ - 6
Sept—2007	0.462874	0.270526	0.266596	3. ‵*^∧^ - 6

**Table 9:** Touristic Shares Summer for Italian Tourists 2008–2014.

Period	Campania	Puglia	Liguria	Zero
Jun—2008	0.332479	0.338985	0.328533	3. ‵*^∧^ - 6
Jul—2008	0.303403	0.421562	0.275032	3. ‵*^∧^ - 6
Aug—2008	0.290416	0.451259	0.258322	3. ‵*^∧^ - 6
Sep—2008	0.457524	0.276158	0.266315	3. ‵*^∧^ - 6
Jun—2009	0.342577	0.3347	0.32272	3. ‵*^∧^ - 6
Jul—2009	0.2966	0.432744	0.270653	3. ‵*^∧^ - 6
Aug—2009	0.27529	0.470632	0.254075	3. ‵*^∧^ - 6
Sep—2009	0.434053	0.298612	0.267332	3. ‵*^∧^ - 6
Jun—2010	0.334029	0.343269	0.322699	3. ‵*^∧^ - 6
Jul—2010	0.296118	0.437483	0.266396	3. ‵*^∧^ - 6
Aug—2010	0.285625	0.471072	0.243301	3. ‵*^∧^ - 6
Sep—2010	0.438586	0.303335	0.258076	3. ‵*^∧^ - 6
Jun—2011	0.336269	0.353042	0.310687	3. ‵*^∧^ - 6
Jul—2011	0.29014	0.451753	0.258104	3. ‵*^∧^ - 6
Aug–2011	0.287526	0.468772	0.243699	3. ‵*^∧^ - 6
Sep–2011	0.448838	0.295442	0.255718	3. ‵*^∧^ - 6
Jun–2012	0.338936	0.35973	0.301331	3. ‵*^∧^ - 6
Jul–2012	0.282261	0.456681	0.261055	3. ‵*^∧^ - 6
Aug–2012	0.27564	0.477004	0.247353	3. ‵*^∧^ - 6
Sep–2012	0.430172	0.335418	0.234406	3. ‵*^∧^ - 6
Jun–2013	0.332694	0.383066	0.284237	3. ‵*^∧^ - 6
Jul–2013	0.28129	0.479494	0.239213	3. ‵*^∧^ - 6
Aug–2013	0.286704	0.483292	0.230001	3. ‵*^∧^ - 6
Sep–2013	0.353113	0.382375	0.264509	3. ‵*^∧^ - 6
Jun–2014	0.325612	0.373144	0.301241	3. ‵*^∧^ - 6
Jul–2014	0.284793	0.470606	0.244598	3. ‵*^∧^ - 6
Aug–2014	0.285578	0.483582	0.230837	3. ‵*^∧^ - 6
Sep–2014	0.359862	0.354163	0.285972	3. ‵*^∧^ - 6

**Fig 8:**
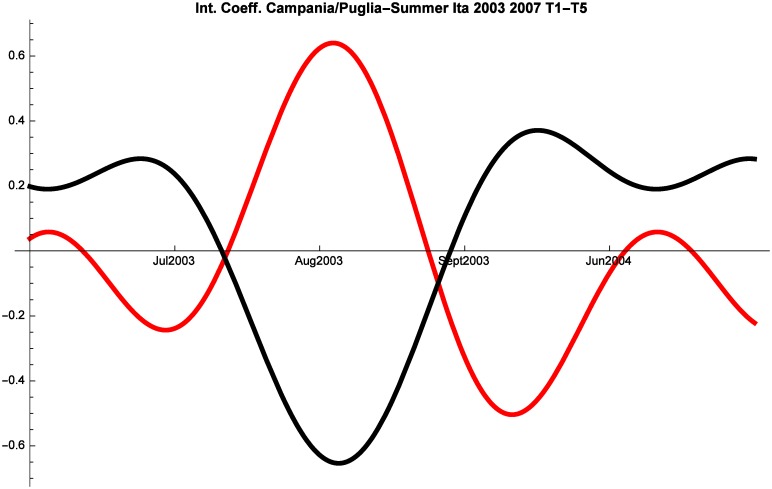
Campania (red) vs Puglia (black) Competitive Roles Summer Italian 2003–2007.

**Fig 9:**
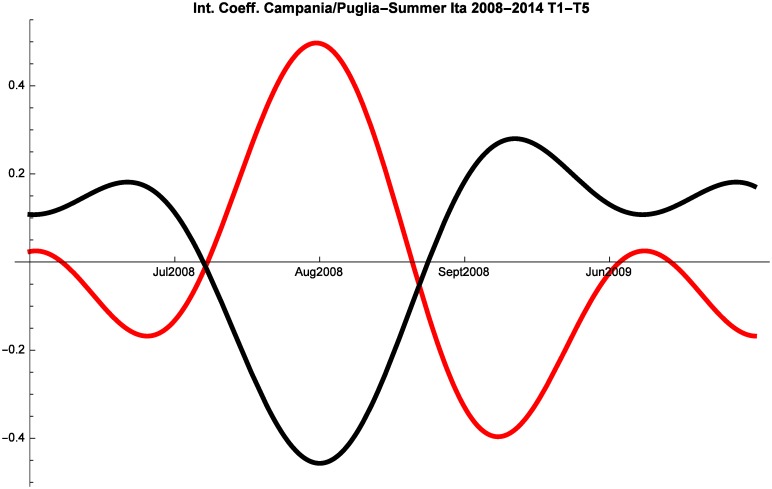
Campania (red) vs Puglia (black) Competitive Roles Summer Italian 2008–2014.

**Fig 10:**
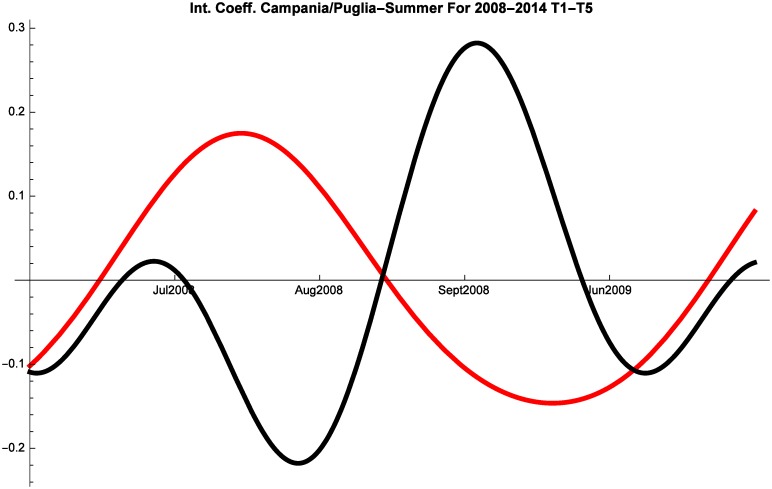
Campania (red) vs Puglia (black) Competitive Roles Summer Foreign 2008–2014.

An additional factor of interest are the interactions between Puglia and Campania shown in Figs 8, 9 and 10. Before the crisis Campania and Puglia are in a relationship of predatory-prey. This suggests that tourists perceived these two regions as close substitutes for summer holidays. Italian tourists maintained this perception also during 2008–2014, whereas during the same years Campania and Puglia managed to develop mutualism for the months of June and July in the segment of foreign tourism.

### Tourists Winter: Italian and Foreign Tourists

**Fig 11:**
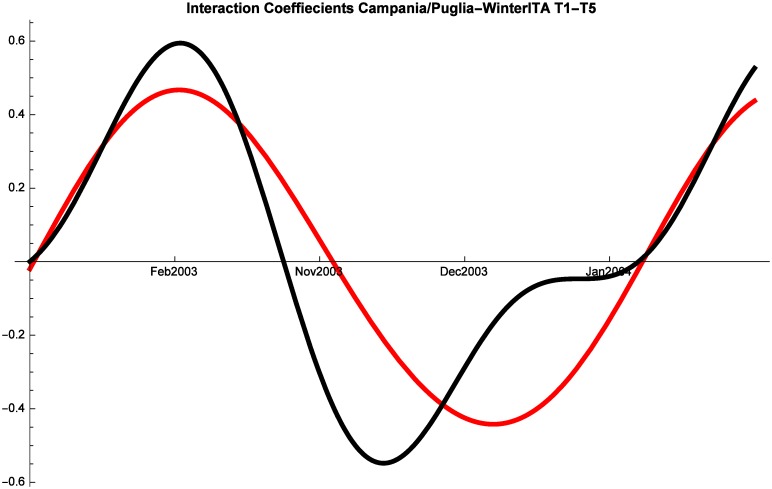
Campania (red) vs Puglia (black) Competitive Roles Winter Italian.

**Fig 12:**
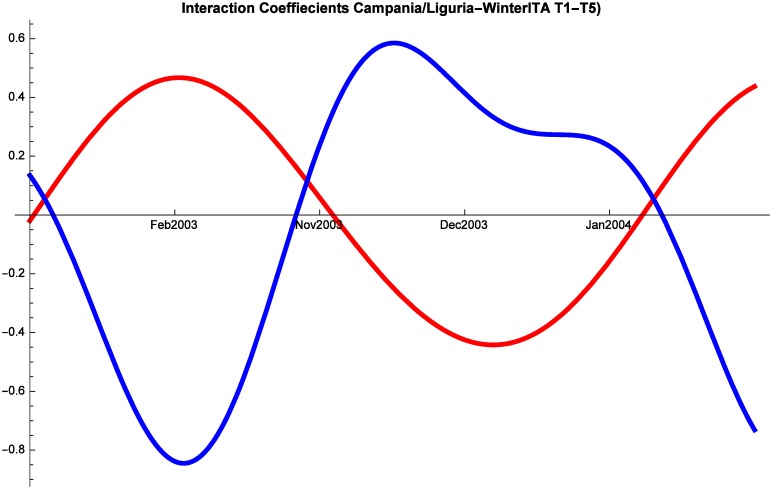
Campania (red) vs Liguria (blue) Competitive Roles Winter Italian.

**Fig 13:**
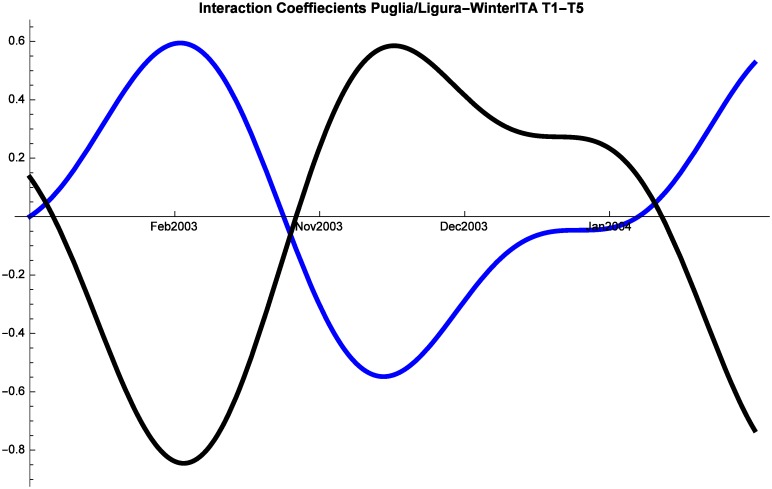
Liguria (blue) vs Puglia (black) Competitive Roles Winter Italian.

As in all the other cases, regular fluctuations characterize tourism dynamics also during winter for both Italian and Foreign tourists. Starting with Italian tourists, we note that Liguria is always in a relationship of predator-prey with both Campania and Puglia (although they alternate as predators and preys), while at times Campania and Puglia proceed in mutualism (Figs 11, 12 and 13). The same dynamics are observed also in the period 2008–2014 and therefore the interactions were not altered by the crisis.

This finding suggests that for tourism not strictly related to the sea, Italian tourists are heavily influenced by their perception of the umbrella brand South Italy as a whole. If the perception of South of Italy deteriorates the tourists opt for locations elsewhere. On the contrary, if the perception improves, all Southern regions benefit from it. In other words, for this niche of the market Southern regions should attempt to pursue a collaborative advantage instead of competing against each other.

Last, let us analyze winter patterns for foreign tourists. Once again, there are constant fluctuations of touristic shares. Interestingly, also for this segment of the market Puglia and Campania show some mutualism, whereas they are almost always in a predator prey relationship with Liguria. These dynamics are not significantly altered by the crisis.

## Discussion

At a general level, the empirical analysis presented in this article leads to four main findings. First, nonautonomous LV models can provide a very accurate fit for tourism dynamics. As shown in Tables 1 and 2, for each of the 12 cases considered the MAPEs and the MSEs indicate that the model can appropriately mimic tourism dynamics. Second, the model identifies nuanced forms of interaction in all of the cases considered, thus suggesting that LV models are a useful tool to investigate tourism dynamics. Third, tourism dynamics present a quasi-fractal pattern. At all the levels analyzed (absolute value, yearly, seasonal, monthly) the data are best approximated by a periodic function. We remark that these fluctuations are very different from the seasonality issue extensively discussed in the literature [38], because they regard touristic shares and not the absolute number of tourists. In other words, these fluctuations indicate that some regions are always relatively stronger than others in some months of the year, whereas in other months they are always relatively weaker. Interestingly, this pattern can be observed also *within* the same season. In other words, even if we “zoom-in” and observe data relative to shorter time intervals, we always observe similar patterns of periodic fluctuations. For example, within the summer season Puglia has a significantly higher touristic share in July and August than in June and September. For Campania the exact opposite holds. This suggests that the negative consequences of seasonality *within* the same season might be more severe for Puglia than for Campania. And indeed, Puglia is relatively stronger in months in which the absolute value of the touristic flow is higher, thus implying a higher variance in touristic presences *within* summer months. Last, for most of the segments analyzed the crisis did not significantly alter competitive dynamics among the destinations considered. Moreover, the few changes observed are related to foreign tourists and have generally produced less competition and more mutualism.

Abandoning the general level, the results presented have some direct implications for the two Southern regions considered.

Campania is unquestionably one of the most beautiful regions in Europe. Besides the breathtaking landscapes and the world-renowned seaside locations, it has a rich and unparalleled history. This is testified also by the 6 sites that UNESCO labelled as World Cultural Heritage. To put it differently, the UNESCO has testified that Campania alone has as much or more cultural heritage than 28 countries included in the category “Europe and North America”. And indeed, there are 28 countries in this category with 6 or less UNESCO sites. Napoli, the main city of the Region, is the jewel of the crown. In the words of Stendhal, without any doubt the most beautiful city of the universe [39]. However, despite an incredible combination of cultural, natural and intangible assets, Campania has never been able to fully exploit its potential in terms of touristic flows [35]. Although a detailed analysis of the causes behind this systematic failure lies outside the scope of this article, some considerations naturally follow from the analysis presented.

First, during both 2003–2007 and 2008–2014 Campania has a much higher touristic share of foreign tourists than Italian tourists. This is especially true for non-summer months like January and February where the difference in the touristic share reaches peaks of around 30%. The most straightforward explanation is that the brand Campania is much weaker within Italian borders than outside. As testified by many studies, the perception that Italians have of Campania is generally very negative. Often, this negative perception is not based on facts. For example, using data collected in 2002 by ISTAT some scholars have estimated the Regional Insecurity Perception Index of each Italian region [40]. Unsurprisingly, Campania is perceived as by far the most dangerous region, with an index that is twice as high as that of the second region (Puglia). It is interesting to combine this finding with the data present on the ISTAT website [41]. In particular, also between 2005 and 2012 Campania was perceived as the most dangerous region in Italy, thus supporting the analysis presented in [40]. However, for each of the 5 years in which data are available (2010–2014) Campania reported crime rate has been between 17% and 20% below national average (see Table 10).

**Table 10:** Reported Crime Rates per 100000 inhabitants between 2010 and 2014 in Campania vs National average.

Year	Italy	Campania	Difference (%)
2010	04333.5	03557.9	0.178978
2011	04550.1	03762.1	0.173183
2012	04734.4	03857.5	0.185219
2013	04801.5	03853.9	0.197355
2014	04627.4	03833.2	0.17163

If this discrepancy between perceived problems and reality is not limited to crime it might have strongly hindered the growth of domestic tourism in Campania. This seems to be especially true for cultural tourism, despite Naples having been defined by the literature on tourism as a superstar art city [42].

Interestingly, Campania has two different competitors for Italian tourists. As stated above, during summer the relationship with Puglia is always of predator-prey. The same pattern can be observed with Liguria during winter months. These relationships hold both during 2003–2007 and during 2008–2014. Instead, during both periods there is some mutualism between Puglia and Campania for winter tourism.

Although it is impossible to determine with certainty the reasons behind these patterns, one tentative explanation can be advanced. In particular, it seems that winter tourism is tied to the reputation of the area in broad terms, and not only as a holiday place. In this sense, it could be that both Puglia and Campania suffer (or benefit) when the reputation of South Italy deteriorates (improves). In other words, when one region does well the brand “South Italy” improves and in turn this benefits also the other. Viceversa, if one region does bad also the other might suffer. This would explain the observed mutualism between the two regions. Also, these findings suggest to start communication campaigns promoting South Italy, not only as a touristic area, but also as a place where the quality of life is at times higher than perceived outside.

On the contrary, summer tourism might be driven by different motives, more strictly tied to the quality of the beaches themselves and on how to reach the location. If this is true, then Puglia and Campania might be perceived as rivals. Here, the lack of adequate connections between Puglia and Campania might prevent developing a relationship of mutualism. For example, there are two fast trains in Italy: Italo and Frecciarossa. The former simply does not go to Puglia and also the latter does not serve Campania and Puglia adequately. Besides a very low number of connections, there is no direct fast train between Napoli and Bari. The fastest solution requires changing in Caserta and takes almost 4 hours. The other options take up to 6 hours. Notably, the distance between Napoli and Bari is only 266Km. To make a comparison, Napoli Roma is 227km and there are countless fast connections that take only 1:10h. The connections between important touristic locations are even worse. Reaching Alberobello from Amalfi (two UNESCO sites separated by 316km) is almost impossible. As of 6/2/2016, according to rome2rio.com the fastest solution takes over 8 hours! Improving connections between Southern Cities and touristic locations seems a crucial step to improve the competitiveness of the area, to favor the growth of Puglia brand outside of Italy and to promote mutualism.

## Conclusion

Lotka–Volterra models are extensively used in almost every field of human knowledge to describe the interaction among different entities. These models are especially useful when this interaction is complex and nuanced, as in the case of tourism. This article constitutes a first application of a *nonautonomous* and *integrable* LV model to tourism research. This model is used to investigate the touristic patterns of three Italian regions: Campania, Puglia and Liguria. The empirical analysis carried out in this article allows drawing a number of conclusions. First, the interactions among touristic destination is very nuanced and inherently dynamic. Touristic destinations not always stand in a relationship of pure competition, but at times engage in mutualism, commensalism, predator-prey etc. Second, the patterns of interactions are very stable. Even if the economy was hit by a severe crisis between 2007 and 2009, the dynamics of interaction among the three regions remained very similar for most of the segments considered. From a methodological perspective, this finding suggests that periodic functions are appropriate to describe touristic dynamics. Third, we find that Campania is relatively weak for Italian tourism, whereas Puglia has a limited appeal outside Italian borders. Fourth, the mutualism between Puglia and Campania is still limited, but for foreign tourism it increased after the crisis. Last, for most segments of the market the competitive interaction among Campania, Puglia and Liguria was not significantly altered by the crisis. An important limitation of this work is the exclusive focus on three regions. Future studies can expand the analysis by including more regions or changing the geographical level considered. For example, this model can be applied to study the interaction among contiguous destinations within the same geographical area (e.g. the islands in the gulf of Napoli), or by studying dynamics at a more macro-level (e.g. aggregates of regions or even countries).
